# Narrative systematic review for autism spectrum disorders screening tools in school settings

**DOI:** 10.1136/bmjopen-2025-105317

**Published:** 2026-01-08

**Authors:** Lucy J McCann, Rinad Bakhti, Nishani Fonseka, Dasha Nicholls, Dougal S Hargreaves, Federica Amati, Antonio Ivan Lazzarino, Ritu Mitra, Krishan Narayan, Alex Weston, Shamini Gnani

**Affiliations:** 1 Department of Twin Research & Genetic Epidemiology, King’s College London, London, UK; 2 Department of Brain Sciences, Faculty of Medicine, Imperial College London, London, UK; 3 School of Medicine, Faculty of Medicine and Health Sciences, Keele University, staffordshire, UK; 4 School of Public Health, Faculty of Medicine, Imperial College London, London, UK; 5 Central and North West London NHS Foundation Trust, London, UK; 6 The Giaroli Centre, London, UK; 7 Institute of Psychology, Psychiatry and Neuroscience, King’s College London, London, UK; 8 Listen to Act, London, UK

**Keywords:** Sensitivity and Specificity, Schools, PUBLIC HEALTH

## Abstract

**Abstract:**

**Objectives:**

Early screening for autism spectrum disorder (ASD) can enhance educational and health outcomes for affected children. This narrative systematic review explores school-based screening tools used around the world to identify children with ASD and explore the differences across socio-demographic groups.

**Design:**

Systematic review of electronic databases (EMBASE, MEDLINE, PsycINFO, Cochrane and Scopus) in October 2024 of papers published between 2011 and 2024.

**Setting:**

Mainstream school-based settings globally.

**Participants:**

Children aged 4–16 years old attending mainstream school.

**Interventions:**

School-based screening tools for ASD, including all types of informant and format of tools reported in eligible studies.

**Primary and secondary outcome measures:**

Primary outcomes included prevalence of screen positives, sensitivity and specificity of the screening tools. Secondary outcomes included participants’ sex, socioeconomic status and ethnicity, and the relation of this to the primary outcomes.

**Results:**

Of 7765 eligible articles, 14 studies were included in this review. We identified eight different school-based ASD screening tools. Study populations ranged from 103 to 16 556 children, with sensitivity and specificity varying by screening tool used, age group, setting and ASD prevalence. The percentage of children screening positive for ASD ranged from 0.7% to 8.5%. Studies were conducted in Europe (n=6), Western Pacific (n=4), the Americas (n=3) and Eastern Mediterranean (n=1) regions. No studies explicitly explored accuracy or validity outcomes based on ethnicity or socioeconomic status. Half of the 14 studies (n=7) reported the sensitivity and specificity of the screening tools; sensitivity ranged from 58% to 94% and specificity from 61% to 100%. There was insufficient evidence to recommend any single ASD screening tool.

**Conclusions:**

ASD screening tools vary widely across the globe, with limited standardisation. Evidence is lacking on how ethnicity and socioeconomic status affect their effectiveness in schools. Given the dearth of scientific evidence in this field, collaboration among educators, researchers and policymakers is needed to establish the evidence base for universal screening, identify optimal tools, coordinate their use and ensure their validation for specific populations.

**STRENGTHS AND LIMITATIONS OF THIS STUDY**

- This study provides a comprehensive narrative synthesis of school-based autism spectrum disorder screening tools globally, using a systematic search strategy across five major databases.
- The study examined a wide range of methodological variables, including informant type, population demographics and test performance metrics such as sensitivity and specificity.
- Heterogeneity in study designs and outcome reporting limited the ability to conduct a formal meta-analysis.
- Sociodemographic differences in screening tool effectiveness could not be fully evaluated due to inconsistent or absent reporting across included studies.

##  Introduction

Autism spectrum disorder (ASD) is one of the most common forms of neurodiversity, with a global prevalence estimated at least 1%.^1^ It is characterised by ‘a persistent inability to initiate and sustain reciprocal social interaction and communication, along with inflexible patterns of behaviour and activities that are clearly atypical or excessive for an individual’s age and sociocultural context’.^2^ In the UK, the incidence of ASD has increased by over 700% over the past two decades.^3^ Over this period, several new ASD screening tools have been developed, although consensus is lacking on the implementation of ASD screening in school settings.^3 4^

The average age of an ASD diagnosis is between 4 and 5 years.^5 6^ However, some children and young people are not diagnosed until after leaving school or even into adulthood.^7^ Those who remain undiagnosed are more likely to face social, emotional and behavioural challenges, poor mental health, school dropout and lower academic achievement.^8^,^10^ As adults, they are at a higher risk of unemployment, mental health issues^11^ and involvement in the criminal justice system, where one in three adults has a neurodiverse condition.^12^ Delayed ASD diagnosis is associated with being female, having an intellectual disability, experiencing depression or growing up in a household where English is not spoken.^13^

Early diagnosis of ASD allows timely use of evidence-based interventions that can help prevent the negative effects of a delayed diagnosis, ultimately improving individuals’ quality of life.^8^,^10^ Screening in schools has been proposed as one route to facilitate earlier detection and diagnosis.^14^ Schools provide a universal setting for screening children, although this approach requires balancing sensitivity (accurately identifying children at risk) and specificity (correctly identifying children not at risk). This balance is crucial for the effectiveness of screening tools.^15^ However, to date, no review summarises the currently available tools used globally. This narrative systematic review aims to examine school-based ASD screening tools used worldwide to identify children aged 4–16 years with ASD and explore the challenges and differences across sociodemographic groups.

## Methods

This systematic review of school-based screening tools was conducted in consultation with a young people’s advisory group, affiliated with the National Institute for Health and Care Research Applied Research Collaboration Northwest London, whose members were aged between 14 and 24 years and based in Northwest London.^16^ This review was designed as a scoping review; as such, it was not eligible for registration with PROSPERO.^17^ Narrative synthesis was prespecified as the data synthesis method given anticipated heterogeneity; however, if sufficient homogeneous data were available, we planned to do a meta-analysis.

### Search Strategy

In April 2022, a search was conducted of the EMBASE, MEDLINE, PsycINFO, Cochrane and Scopus electronic databases using relevant key terms related to school children, screening tools and neurodiversity. This search was repeated in October 2024 to identify any additional articles. The full search terms and strategy are detailed in online supplemental tables 1 and 2.

An initial basic search strategy was carried out in MEDLINE and PsycINFO to identify relevant keywords from the titles and abstracts of pertinent manuscripts. Each term was mapped to a PICO (population, intervention, control and outcome) framework. The strategy was then reviewed by academic and clinical colleagues in relevant healthcare fields. A librarian cross-checked the final search strategy to optimise the results and adapted it for each individual database. No manual searches were conducted.

### Eligibility criteria

Articles were included if they used a school-based screening tool for ASD in children aged 4–16 years, as full-time school education is not mandatory outside of this age range in many countries (table 1). Only studies involving and reporting outcomes of participants aged 4–16 years were included.

**Table 1:** Eligibility criteria

	Inclusion criteria	Exclusion criteria
Population	School children attending mainstream schools aged 4–16 years.	Children and young people aged below 4 years and above 16 years.
Intervention	Screening tool for ASD.	Screening tools for neurodiverse conditions other than ASD.
Control	Not applicable.	
Outcome	Screen positive ASD prevalence, sensitivity, specificity and informant of tool.	Other outcome measures of screening or outcomes related to other neurodiverse or mental health conditions.
Setting	School-based.	Screening conducted in non-school settings eg, healthcare or community, or in a non-mainstream (or special) school setting.
Publication	All global studies based on primary research, systematic reviews and meta-analyses. Available in English.	Abstracts, posters, editorials, letters, policy documents, education guidelines, pilot studies and grey literature. Not available in English.
	Published from 2010 onwards.	Published before 2010.

A summary of inclusion and exclusion criteria used to identify eligible studies.

ASD, autism spectrum disorder.

Studies published after 2010 were included to capture recent advancements in ASD screening methods. Systematic reviews and meta-analyses were excluded, as were all other types of research papers, such as book chapters, conference abstracts and grey literature. We restricted inclusion to full-text articles to ensure that there was sufficient methodological detail, and grey literature was excluded as it is not subjected to peer-review process.

### Study selection

All articles identified through the search strategy were compiled in the Covidence systematic review software.^18^ Duplicates were removed, and the titles and abstracts of relevant articles were screened by two independent researchers. Full-text screening was then performed to assess eligibility. The reference lists of all included sources were also reviewed to identify additional studies. Any disagreements were resolved through consensus with a third researcher.

### Data extraction and synthesis

Data were independently extracted by two researchers using Excel. Information collected included the author, year, country, study population, screening tool used, informant, prevalence of ASD screen positives, sensitivity and specificity, and participants’ sex, socioeconomic status and ethnicity. For studies that included both eligible and ineligible subpopulations such as screening in both public schools and special needs schools, we extracted only the data that met our inclusion criteria. No additional or missing data were requested from the authors of the included papers. A narrative synthesis of the eligible studies was presented, alongside a summary table of the identified screening tools.

## Results

**Figure 1:**
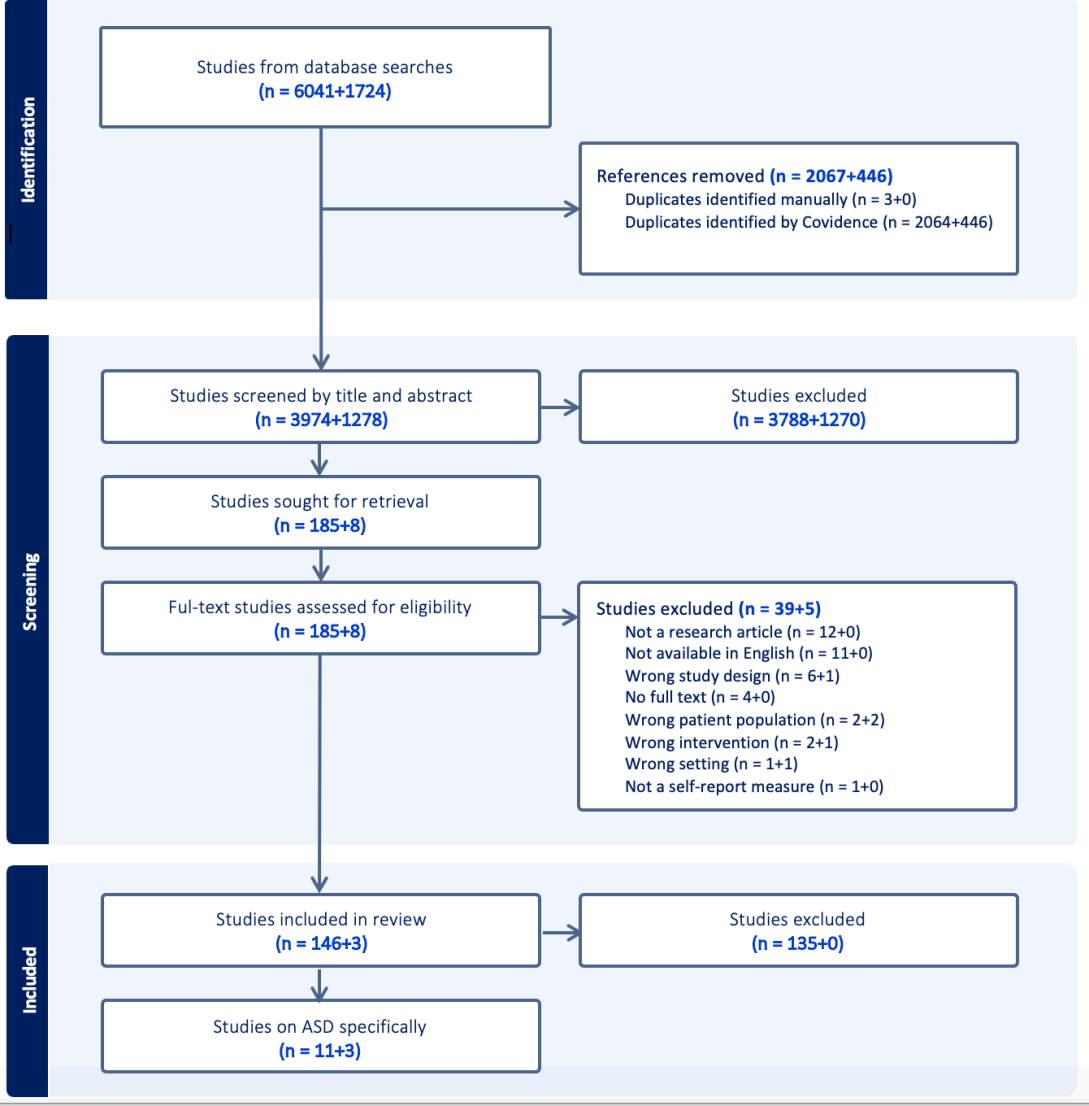
PRISMA flow diagram of study search and selection. ASD, autism spectrum disorder; PRISMA, Preferred Reporting Items for Systematic Reviews and Meta-Analyses.

Figure 1 shows the Preferred Reporting Items for Systematic Reviews and Meta-Analyses flow diagram for the search strategy. Numbers of studies are displayed for the two screening points as X+X. A total of 6041 articles were identified in the initial search and a further 1724 were added in the updated search. After full-text screening, 14 articles were selected for inclusion in this review. A meta-analysis was not possible due to the heterogeneity of the study methods and results. The sample sizes ranged from 103 to 16 556 children, with 9 studies including over 1000 participants.

Table 2 summarises the 14 studies included in this review. These studies were conducted across various WHO regions^19^: Europe (n=6), Western Pacific (n=4), the Americas (n=3) and Eastern Mediterranean (n=1).

**Table 2:** Summary of school-based autism spectrum disorders (ASD) screening studies identified in review (N=14)

	Author (year)	Country	Study population (N)	Sex distribution	Age group (years)	Screening tool used (language)	Screening ASD positive (%); screening by sex; sensitivity and specificity; prevalance (if reported)
1	Alshaban *et al* (2019)^23^	Qatar	9074*	4960 girls, 3716 boys	5–12	Social Communication Questionnaire (SCQ) (parent) (Unspecified)	8.5% screen positive; boys had higher screen positive (9.6%, p<0.003); NA Prevalence diagnosis: 1.1%
2	Cheon *et al* (2016)^33^	Korea	790	378 girls, 412 boys	7–12	Social Responsiveness Scale (SRS) (parent) Autism Spectrum Screening Questionnaire (ASSQ) (parent) (Korean)	SRS: NA; boys had higher SRS scores (p<0.001); 80% sensitivity and 76% specificity (boys); 92% sensitivity and 87% specificity (girls) No separate results for ASSQ. ASSQ score was significantly associated with SRS score (p<0.01).
3	Fombonne *et al* (2012)^20^	Mexico	563	185 girls, 378 boys	4–13	Social Responsiveness Scale (SRS) (parent and teacher) (Spanish)	NA; no sex difference (p=0.90); SRS (parent): 93% sensitivity & 93% specificity; SRS (teacher): 94% sensitivity and 84% specificity
4	Fombonne *et al* (2016)^34^	Mexico	4195 (parent) 2761 (teacher) 4431 (parent or teacher)	2120 girls, 2074 boys (parent) 1402 girls, 1358 boys (teacher)	8	Social Responsiveness Scale (SRS) (parent and teacher) (Spanish)	1.0% screen positive; boys had higher scores (p<0.001); NA
5	Hashmi *et al* (2021)^35^	Malaysia	653	336 girls, 317 boys	6–12	Autism Spectrum Quotient Children’s Version (AQ-Child) (parent) (Malay)	NA; NA; 93% sensitivity and 99% specificity
6	Morales-Hidalgo *et al* (2017)^36^	Spain	2660 (1359 aged 4–5, 1301 aged 10–11)	1342 girls, 1318 boys	4–5 and 10–12	EDUTEA Questionnaire (teacher/ parent) Childhood Asperger Syndrome Test (CAST) (parent) (Unspecified)	EUDTEA: NA; boys have higher scores (p=0.001); 87% sensitivity and 91% specificity No separate results for CAST.
7	Morales-Hidalgo *et al* (2018)^25^	Spain	1407	767 girls, 640 boys	10–12†	EDUTEA Questionnaire (teacher) Childhood Autism Spectrum Test (CAST) (parent) (Spanish)	EDUTEA: 6.2% screen positive; no difference by sex (p>0.05); NA No separate results for CAST as used as an adjunct. Estimated population prevalence: 1.0%
8	Rasga *et al* (2023)^24^	Portugal	13 690	NA	7–9	Teacher Nomination Form (teacher) (Portuguese)	0.7% screen positive; boy-to-girl ratio 2.9:1 diagnosed; NA Estimated population prevalence: 0.5%
9	Scattoni *et al* (2023)^37^	Italy	16 293	NA	7–9	Social Communication Questionnaire (SCQ) - Lifetime (parent) (Unspecified)	2.4% screen positive; NA; NA Estimated population prevalence: 1.4% (boy-to-girl ratio of 4.4:1)
10	Schanding *et al* (2012)^21^	USA	3375	NA	4–17	SCQ - Current (teacher) SCQ - Lifetime (parent) Social Responsiveness Scale (SRS) (teacher and parent) (English)	NA; NA; SCQ Current: 60% sensitivity and 95% specificity; SCQ Lifetime: 75% sensitivity and 100% specificity. SRS (teacher): 69% sensitivity and 95% specificity; SRS (parent): 80% sensitivity and 99% specificity
11	Sun *et al* (2014)^38^	China	682	322 girls, 360 boys	6–11	Childhood Autism Spectrum Test (CAST) (parent) (Mandarin)	NA; boys had higher scores (p<0.001); NA
12	Sun *et al* (2014)^22^	China	103	45 girls, 58 boys	4–11	Childhood Autism Spectrum Test (CAST) (parent) Clancy Autism Behaviour Scale (CABS) (parent) (Mandarin)	NA; NA; CAST: 89% sensitivity and 80% specificity; CABS: 58% sensitivity and 84% specificity CAST better than CABS in screening for ASD (p=0.0002)
13	Sun *et al* (2014)^39^	UK	3329*	1663 girls, 1658 boys	5–10	Childhood Autism Spectrum Test (CAST) (parent) (English)	NA; boys scored higher than girls (p=0.001); NA
14	Zirakashvili *et al* (2022)^40^	Georgia	16 556	NA	8	Autism Spectrum Screening Questionnaire (ASSQ) (parent and teacher) (Georgian)	NA; NA; ASSQ (parent): 83% sensitivity and 41.8% specificity; ASSQ (teacher): 83% sensitivity and 46.8% specificity; ASSQ (combined): 79.2% sensitivity and 61.1% specificity

Summary of all school-based ASD screening studies identified in the review including data extracted.

* Potential missing data within this study as population greater than figures presented for boys and girls combined.

† A group aged 3–5 was also included in the study but has been excluded from this analysis as this age range goes outside of the inclusion criteria

DSM5, Diagnostic and Statistical Manual of Mental Disorders, 5 edition; NA, not available.

### Screening tools identified

Half of the 14 studies (n=7) reported the sensitivity and specificity of the screening tools; sensitivity ranged from 58% to 94% and specificity from 61% to 100%. We identified eight different school-based ASD screening tools (table 3). The Social Responsiveness Scale (parent and teacher) had the highest reported sensitivity (94% and 93%, respectively) with optimal cut-offs of 52 on the parent Social Responsiveness Scale and of a cut-off of 50 on the teacher scale,^20^ when used in a sample of Mexican children aged 4–13 years (of which 200 had pervasive developmental disorder (PDD) and 363 were typically developing). The Social and Communication Questionnaire : Lifetime (parent)^21^ had the highest specificity (100%); however, this was then associated with a lower sensitivity (75%).

**Table 3:** Description of school-based autism spectrum disorder (ASD) screening tools identified in review

Name of ASD screening tool		Age group (years)	Number of items	Country of authors origin	Informant	Non-English Languages within study	Reference
1	Autism Spectrum Quotient Children’s Version (AQ-Child)	4–11	50	UK	Parent	Malay	(Auyeung *et al*, 2008)^41^
2	Autism Spectrum Screening Questionnaire (ASSQ)	7–16;	27	Sweden	Parent/Teacher	Georgian	(Ehlers *et al*, 1999)^42^
3	Clancy Autism Behaviour Scale (CABS)	Pre-school	14	Australia	Parent	Mandarin	(Clancy *et al*, 1969)^43^
4	Childhood Autism Spectrum Test (CAST)*	4–11	37	UK	Parent	Mandarin	(Scott *et al*, 2002)^44^
5	Social Communication Questionnaire (SCQ): current and lifetime†	4–21	40	USA	Parent/Teacher	None	(Rutter *et al*, 2003)^45^
6	Social Responsiveness Scale (SRS): first and second edition	2.5–18	65	USA	Parent/ Teacher	Korean/Spanish	(Constantino, 2012)^46^
7	Teacher Nomination Form	5–11	6	USA	Teacher	Portuguese	(Hepburn *et al*, 2017)^47^
8	EDUTEA Questionnaire	3–12	11	Spain	Teacher	Spanish	(Morales-Hidalgo *et al*, 2017)^36^

Summary of all the ASD screening tools identified in this review, with background information.

* Formerly the Childhood Asperger Syndrome Test

† Formerly the Autism Screening Questionnaire (ASQ)

We identified eight different school-based ASD screening tools (table 3). The Childhood Autism Spectrum Test was the most used tool to screen for ASD (n=5), followed by the Social Responsiveness Scale (n=4) and the Social and Communication Questionnaire (n=3). Of the five studies that included the Childhood Autism Spectrum Test, only one reported sensitivity and specificity (89% and 80%, respectively).^22^ Nine studies used a single screening tool, and five used multiple screening tools within the same population. No studies used screening tools in different stages to screen for ASD.

Each ASD screening tool varied significantly in the number of items/questions included (table 3), ranging from 6 items (Teacher Nomination Form) to 65 items (Social Responsiveness Scale). Of the eight tools, 75% (n=6) were developed in English-speaking countries: 38% in the USA (n=3), 25% in the UK (n=2), and 13% in Australia (n=1), with the remaining two from Spain (13%, n=1) and Sweden (13%, n=1).

Within these studies, almost all of the tools (88%, n=7) were translated into languages other than English. For example, the Social Responsiveness Scale was translated into Korean and Spanish. Screening tools in school settings used parents and teachers as informants in different combinations including only teachers (n=1), only parents (n=7) and a combination of both (n=6). No studies used self-report screening tools.

Only five studies reported the percentage of screen-positive ASD screening tests within the populations assessed, with figures ranging from 0.7% to 8.5% within the studied population.^23^ ^24^

### Differences by sociodemographic groups

Of the 14 studies, 10 (71%) reported outcomes based on the sex of participants. Of these, eight found that boys had higher scores for screening percentage, estimated prevalence or diagnosis prevalence than girls, with the remaining two finding no statistically significant sex difference in screening results.

Notably, Morales-Hidalgo *et al*^25^ was the only study that conducted an in-depth analysis of socio-demographic factors (including socioeconomic status, environment and ethnicity), and found that sex was the only sociodemographic variable that was associated with an ASD diagnosis (p<0.02) for both estimated prevalence and direct prevalence.

## Discussion

### Summary of evidence

To our knowledge, this is the first review that summarises the currently available ASD tools used globally in school settings. Eight different school-based ASD screening tools were identified across 11 different countries within 14 published studies. The percentage of screen-positive results for ASD ranged from 0.7% to 8.5%. The majority of studies reported higher screen-positive results in boys; however, there was limited consideration of other sociodemographic factors and their interplay with screening results. The interpretation of the outcomes was limited by the heterogeneity of reported outcomes.

It is important to acknowledge that only 3 of the 11 countries are classified as low-income and middle-income status—Mexico, Georgia and Malaysia. A 2019 review on ASD screening tools in low-income and middle-income countries by Marlow *et al* highlighted that the optimal tools within low-resource settings may differ due to a range of factors such as affordability and ease of use.^4^ The same considerations are likely to apply to non-Western settings.

Only half of the included studies reported sensitivity and specificity, limiting the ability to draw firm conclusions about the performance of screening tools across different settings. While screening can support earlier identification of ASD, it is important to acknowledge ongoing uncertainty around its benefits and limitations.^26 27^ Screening tools should be seen as one component within a broader diagnostic pathway, rather than standalone assessments. Importantly, while early diagnosis and intervention are strongly associated with improved outcomes, it remains unclear whether early universal screening programmes reliably deliver earlier diagnosis which translates into these long-term outcomes for individuals.^8^,^10^ Previous research, including a cohort study by Russell *et al*^26^ and a commentary by Szatmari,^27^ has highlighted concerns about the evidence base for universal screening, particularly regarding the impact of early diagnosis on later functioning.

Limited reporting of sociodemographic characteristics at both the baseline and outcome level was also identified. Adequate reporting of these factors is essential to understand the differential detection rate of screening tools across characteristics that are known to stratify health outcomes and opportunities.^28^ Without this, screening tools have the potential to widen inequalities within populations. Addressing this through screening is vital, as recent work has shown that a diagnosis of ASD is less likely to be made within ethnic minorities and families from lower socioeconomic groups.^29^ Careful recording and consideration of health inequalities has the potential to not only prevent worsening disparities but also improve equity. A previous pilot study conducted in Bradford, UK, demonstrated the potential to address equity of access through school-based screening, whereby all nine of the children newly diagnosed in the pilot study were from ethnic minorities.^29^

### Strengths and limitations

Some strengths and limitations must be acknowledged. Our comprehensive search strategy across a range of databases identified 14 studies across 11 different countries, and over 70 000 children and young people in total. These studies reported a range of outcomes, including the percentage of screen-positive, sensitivity, specificity and comparison of results by sex. However, other educational databases, such as education full text, might have offered access to additional relevant research studies. Similarly, we did not undertake supplementary searches such as contacting authors or reviewing manuals of individual screening tools, which may have identified additional validation studies. Our search did not include all possible educational terms (eg, all possible variations on school education, college) or variations of neurodiversity-related terms (eg, Asperger’s syndrome), which may limit comprehensiveness. However, evidence on population-level medical screening is mainly published in medical and public health journals, so the impact is likely minimal. The heterogeneity of the study outcomes and populations included prevented the possibility of a meta-analysis. Our approach of only including ASD-specific screening tools may have also missed literature using a combined approach to neurodiversity, such as studies like Kaur *et al* which aimed to identify symptom-based clusters using behavioural characteristics.^30^ However, this broad approach is likely to produce more false positives.

Another limitation is the time frame of the studies in relation to the COVID-19 pandemic—only four were conducted post-2020. The pandemic significantly influenced ASD assessment and referral pattern,^31^ with evidence suggesting an increase in referral patterns following lockdowns. While the reasons for this rise are likely multifactorial, the disruptions to social development may have influenced the presentation and identification of ASD traits. Additionally, increasing ASD awareness and education within schools over the past decade may have affected the interpretation and scoring of screening tools. The extent to which these factors may have influenced the performance or validity of screening tools in recent years remains unclear.

It is important to note that not all studies compared the screening tool to a diagnostic gold standard, limiting our ability to comment on sensitivity and specificity within some studies. Instead, several studies only reported the proportion of participants who screened positive, which can be influenced by both the true prevalence of autism in the population and the characteristics of the test itself. We have aimed to use consistent terminology throughout the paper, distinguishing between detection rates or proportion screening positive, and true sensitivity or specificity.

### Implications for policymakers

Recommendations for universal ASD screening in schools involve a complex balance of risks and benefits. Screening needs to be accurate, appropriate, timely and be accompanied by accessible, effective and acceptable interventions; otherwise, children may be labelled with a ‘diagnosis’ without care and provision. ASD screening protocols and care planning are needed given the rise in diagnoses. This should incorporate prescreening discussions with families, particularly in ethnic minority groups, where stigma remains a barrier and concerns may exist regarding diagnosis-related stigma and potential impacts on educational opportunities. Addressing these issues through education and awareness is essential to improving engagement and accessibility. In this review, we did not find evidence that any one screening tool was preferred or outperformed others; each has its strengths and limitations, and the lack of data on sensitivity and specificity for many of the tools limits their potential implementation at a policy level.

There is still debate regarding the appropriate setting for universal implementation of ASD screening. Many Organisations for Economic Co-operation and Development (OECD) countries have implemented national screening tests of developmental disorders including ASD because of a high prevalence of the condition. The American Academy of Paediatrics (AAP) recommends universal ASD screening at routine paediatric visits alongside screening for broad developmental conditions.^32^ At the societal level, financial and infrastructural costs of screening need to be considered. Here, school-based screening may be beneficial and potentially mitigate any potential harms from screening, such as ostracisation, social isolation, poor educational attainment and false diagnosis.

### Conclusions

There is significant variation in ASD screening tools used in schools, with limited data on their sensitivity and specificity. The lack of evidence on how ethnicity and socioeconomic status affect the accuracy of the tools raises concerns about their potential to widen existing health inequalities. Collaboration among healthcare workers, educators, researchers and policymakers is crucial for establishing the evidence base for universal screening, selecting the most appropriate screening tools, determining the optimal screening locations, ensuring these tools are validated for the populations they are intended to serve and evaluating outcomes of screening implementation.

## Supplementary material

10.1136/bmjopen-2025-105317 online supplemental file 1

## Data Availability

No data are available.
