# Supplementary material for: Narrative systematic review for autism spectrum disorders screening tools in school settings
Source: BMJ Open. 2026 Jan 8;16(1):e105317. doi: 10.1136/bmjopen-2025-105317 (PMC13059882; doi:10.1136/bmjopen-2025-105317)
Supplement: online supplemental file 1 [file bmjopen-16-1-s001.docx]

# Supplemental Materials

**Supplementary Table 1:** Search Terms used

|  | **Main Concept** | **Keyword** | **Medical Subject Headings** |
| --- | --- | --- | --- |
| **P** | School children aged 4-16 years  School setting | classroom*.mp.  pupil*.mp.  student*.mp. | Schools  Child |
| **I** | Screening tools | screen*.mp.  detect*.mp.  tool*.mp. | exp Mass Screening/ |
| **C** | N/A | N/A | N/A |
| **O** | Neurodiversity | autis*.mp.  attention deficit hyperactive disorder*.mp.  ADHD.mp.  ASD.mp  Autistic disorder.m.p  dyslexia.mp. | exp Autism Spectrum Disorder/  exp Attention Deficit Disorder with Hyperactivity/  Autistic Disorder/  exp Dyslexia/  exp Specific Learning Disabilities/ |

**Supplementary Table 2:** Search Strategy used

Search strategies for all five databases (Ovid MEDLINE, APA PsycInfo, Embase, Cochrane Library, and Scopus) are provided below, exactly as run in October 2024:

Ovid MEDLINE(R) ALL <1946 to October 04, 2024>

1 student*.mp. 433424

2 limit 1 to yr="2010 -Current" 282389

3 school*.mp. 407285

4 limit 3 to yr="2010 -Current" 226356

5 *Schools/ 23196

6 limit 5 to yr="2010 -Current" 11484

7 2 or 4 or 6 428672

8 screen*.mp. 1151017

9 limit 8 to yr="2010 -Current" 780970

10 *Mass Screening/ 58510

11 limit 10 to yr="2010 -Current" 24802

12 detect*.mp. 3023172

13 limit 12 to yr="2010 -Current" 1773370

14 9 or 11 or 13 2369488

15 autis*.mp. 77352

16 limit 15 to yr="2010 -Current" 61939

17 *Autistic Disorder/ 23548

18 limit 17 to yr="2010 -Current" 13089

19 exp Autism Spectrum Disorder/ 46295

20 limit 19 to yr="2010 -Current" 33483

21 *Attention Deficit Disorder with Hyperactivity/ 30917

22 limit 21 to yr="2010 -Current" 19677

23 ADHD.mp. 34874

24 limit 23 to yr="2010 -Current" 26533

25 dyslex*.mp. 11567

26 limit 25 to yr="2010 -Current" 4926

27 *Dyslexia/ 7424

28 limit 27 to yr="2010 -Current" 3072

29 16 or 18 or 20 or 22 or 24 or 26 or 28 92021

30 7 and 14 and 29 1256

31 limit 30 to abstracts 1250

32 limit 31 to ("preschool child (2 to 5 years)" or "child (6 to 12 years)" or "adolescent (13 to 18 years)") 821

33 limit 32 to yr="2022 -Current" 188

APA PsycInfo <1806 to September 2024 Week 5>

1 student*.mp. 769245

2 limit 1 to yr="2010 -Current" 352146

3 school*.mp. 554208

4 limit 3 to yr="2010 -Current" 272823

5 *Schools/ 17815

6 limit 5 to yr="2010 -Current" 9386

7 2 or 4 or 6 464338

8 *Screening/ 7430

9 limit 8 to yr="2010 -Current" 3873

10 screen*.mp. 157770

11 limit 10 to yr="2010 -Current" 107534

12 detect*.mp. 178512

13 limit 12 to yr="2010 -Current" 109214

14 9 or 11 or 13 204069

15 autis*.mp. 75016

16 limit 15 to yr="2010 -Current" 53140

17 autistic disorder.mp. 12889

18 limit 17 to yr="2010 -Current" 5141

19 *Autism Spectrum Disorders/ 54626

20 limit 19 to yr="2010 -Current" 39671

21 *Attention Deficit Disorder with Hyperactivity/ 29355

22 limit 21 to yr="2010 -Current" 18331

23 ADHD.mp. 36700

24 limit 23 to yr="2010 -Current" 24406

25 *Dyslexia/ 7201

26 limit 25 to yr="2010 -Current" 3307

27 dyslexia.mp. 12022

28 limit 27 to yr="2010 -Current" 5009

29 16 or 18 or 20 or 22 or 24 or 26 or 28 79336

30 7 and 14 and 29 1833

31 limit 30 to abstracts 1833

32 limit 31 to (160 preschool age <age 2 to 5 yrs> or 180 school age <age 6 to 12 yrs> or 200 adolescence <age 13 to 17 yrs>) 1233

33 limit 32 to yr="2022 -Current" 190

Embase Classic+Embase <1947 to 2024 October 04>

1 student*.mp. 673685

2 limit 1 to yr="2010 -Current" 484038

3 school*.mp. 954403

4 limit 3 to yr="2010 -Current" 587782

5 *Schools/ 24371

6 limit 5 to yr="2010 -Current" 12349

7 2 or 4 or 6 960105

8 screen*.mp. 1879357

9 limit 8 to yr="2010 -Current" 1336289

10 *mass screening/ 26112

11 limit 10 to yr="2010 -Current" 7810

12 detect*.mp. 4056428

13 limit 12 to yr="2010 -Current" 2569795

14 9 or 11 or 13 3620152

15 autis*.mp. 115085

16 limit 15 to yr="2010 -Current" 93810

17 Autism spectrum disorder.mp. 39199

18 limit 17 to yr="2010 -Current" 38130

19 *autism/ 59283

20 limit 19 to yr="2010 -Current" 47354

21 exp attention deficit hyperactivity disorder/ 16500

22 limit 21 to yr="2010 -Current" 16483

23 ADHD.mp. 51044

24 limit 23 to yr="2010 -Current" 40629

25 dyslex*.mp. 14752

26 limit 25 to yr="2010 -Current" 7295

27 *dyslexia/ 8394

28 limit 27 to yr="2010 -Current" 3303

29 16 or 18 or 20 or 22 or 24 or 26 or 28 138993

30 7 and 14 and 29 4203

31 limit 30 to abstracts 4175

32 limit 31 to (preschool child <1 to 6 years> or school child <7 to 12 years> or adolescent <13 to 17 years>) 3079

33 limit 32 to yr="2022 -Current" 887

**Cochrane**

Search Name: neurodiversity-updated1

Date Run: 06/10/2024 00:04:29

Comment:

ID Search Hits

#1 MeSH descriptor: [Schools] explode all trees 5873

#2 (student*):ti,ab,kw (Word variations have been searched) with Cochrane Library publication date Between Jan 2010 and Jun 2022 33300

#3 school* with Cochrane Library publication date Between Jan 2010 and Jun 2022 90956

#4 #1 OR #2 OR #3 with Cochrane Library publication date Between Jan 2010 and Jun 2022 114894

#5 MeSH descriptor: [Mass Screening] explode all trees 6079

#6 screening with Cochrane Library publication date Between Jan 2010 and Jun 2022 60789

#7 detect* with Cochrane Library publication date Between Jan 2010 and Jun 2022 75802

#8 #5 OR #6 OR #7 126881

#9 autis* with Cochrane Library publication date Between Jan 2010 and Jun 2022 4025

#10 MeSH descriptor: [Autistic Disorder] explode all trees 1639

#11 ASD with Cochrane Library publication date Between Jan 2010 and Jun 2022 2322

#12 ADHD with Cochrane Library publication date Between Jan 2010 and Jun 2022 4357

#13 MeSH descriptor: [Attention Deficit Disorder with Hyperactivity] explode all trees 3866

#14 dyslex* with Cochrane Library publication date Between Jan 2010 and Jun 2022 347

#15 MeSH descriptor: [Dyslexia] 5 tree(s) exploded 399

#16 #9 OR #10 OR #11 OR #12 OR #13 OR #14 OR #15 11304

#17 #4 AND #8 AND #16 with Cochrane Library publication date Between May 2022 and Oct 2024 6

**Scopus-**

( ( TITLE-ABS-KEY ( student* ) ) OR ( TITLE-ABS-KEY ( school* ) ) ) AND ( ( TITLE-ABS-KEY ( screening ) ) OR ( TITLE-ABS-KEY ( detect ) ) ) AND ( ( TITLE-ABS-KEY ( autis* ) ) OR ( TITLE-ABS-KEY ( "attention deficit disorder with hyperactivity" ) ) OR ( TITLE-ABS-KEY ( "autistic disorder" ) ) OR ( TITLE-ABS-KEY ( adhd ) ) OR ( TITLE-ABS-KEY ( asd ) ) OR ( TITLE-ABS-KEY ( dyslex* ) ) ) AND ORIG-LOAD-DATE AFT 20220817 AND ( LIMIT-TO ( PUBYEAR , 2022 ) OR LIMIT-TO ( PUBYEAR , 2023 ) OR LIMIT-TO ( PUBYEAR , 2024 ) )

453
