# Supplementary material for: Narrative systematic review for autism spectrum disorders screening tools in school settings
Source: BMJ Open. 2026 Jan 8;16(1):e105317. doi: 10.1136/bmjopen-2025-105317 (PMC13059882; doi:10.1136/bmjopen-2025-105317)
Supplement: Supplementary file 2 [file bmjopen-2025-105317.reviewer_comments.pdf]

PEER REVIEW HISTORY

BMJ Open publishes all reviews undertaken for accepted manuscripts. Reviewers are asked to complete a checklist review form and are provided with free text boxes to elaborate on their assessment. These free text comments are reproduced below.

ARTICLE DETAILS

Title (Provisional)

A narrative systematic review for Autism Spectrum Disorders screening tools in school settings

Authors

McCann, Lucy Jane; Bakhti, Rinad; Fonseka, Nishani; Nicholls, Dasha; Hargreaves, Dougal S; Amati, Federica; Lazzarino, Antonio Ivan; Mitra, Ritu; Narayan, Krishan; Weston, Alex; Gnani, Shamini

VERSION 1 - REVIEW

**Reviewer**	**1**
**Name**	**Bujnowska-Fedak, Maria**
**Affiliation**	**Wroclaw Medical University**
**Date**	**14-Jun-2025**
**COI**	**None**

Autism spectrum disorder (ASD) is a category of neurodevelopmental disorders characterized by challenges concerning social skills, speech development and behavior. Early diagnosis of these disorders allows for the initiation of early therapy, which is crucial for the child’s further functioning in society. This narrative systematic review aims to examine school-based ASD screening tools used worldwide to identify children aged 4 to 16 years with ASD and explore the challenges and differences across socio-demographic groups.

The paper has a clear friendly structure (Introduction, Methods, Results, Discussion with Strengths and Limitations section, Conclusion) and is easy to read. The subject is interesting and useful and raises the important issue of early diagnosis of ASD which can enhance educational and health outcomes for affected children. The introduction is quite informative and the topic is explained in detail. The manuscript provides a comprehensive narrative synthesis of school-based ASD screening tools, using a systematic search strategy across five major databases. The text has been supplemented with one figure and three tables in the main manuscript body and two tables in the supplementary materials, and enriched with 41 relevant references.

However there are a few issues which should be corrected and needs further explanation:

1. The number of identified school-based ASD screening tools is different in the abstract, where seven tools are mentioned, and in the main text, where eight tools were identified and analyzed. This requires correction.

2. The discussion needs to be expanded and take on a broader perspective. It also seems inconsistent and contradicts earlier obvious statements in some parts.

In Discussion (page 15) in lines 51-56 there is a sentence: ‘Importantly, it remains unclear whether early detection through screening improves long-term outcomes for individuals living with ASD’. Do you really agree with this statement? There is strong evidence that early diagnosis of ASD is crucial and can positively impact a child’s later development.

For example, look at the references below:

Smith T, Klorman R, Mruzek DW. Predicting Outcome of CommunityBased Early Intensive Behavioral Intervention for Children with Autism. J Abnorm Child Psychol. 2015;43:1271–82.

Orinstein AJ, Helt M, Troyb E, Tyson KE, Barton ML, Eigsti IM, et al. Inter- vention for optimal outcome in children and adolescents with a history of Autism. J Dev Behav Pediatr. 2014;35:247–56.

MacDonald R, Parry-Cruwys D, Dupere S, Ahearn W. Assessing progress and outcome of early intensive behavioral intervention for toddlers with autism. Res Dev Disabil. 2014;35:3632–44.

Sobieski, M., Sobieska, A., Sekułowicz, M. et al. Tools for early screening of autism spectrum disorders in primary health care – a scoping review. BMC Prim. Care 23, 46 (2022). https://doi.org/10.1186/s12875-022-01645-7.

Furthermore, this statement contradicts your previous statement in the Introduction (page 5, lines 42-44): ‘Screening enables earlier evidence-based interventions that can help prevent the negative effects of a delayed ASD diagnosis, ultimately improving individuals' quality of life’.

**Reviewer**	**2**
**Name**	**Marshall, David**
**Affiliation**	**Lime Trees CAMHS , Research Team**
**Date**	**07-Aug-2025**
**COI**	**None**

The article is a systematic review of screening tools for autism used in educational settings. It appears to have been well conducted and used appropriate methodology, however there are a number of areas where I have concerns. Most of these are fairly minor and a result of not adequately reviewing the paper before submitting but my two main concerns are the

breadth of the searches and the inaccuracy of reporting some of the results. I'll detail all my concerns by section below.

Abstract

1. I actually think this is well written for the most part. My only concern is that the PROSPERO regitration ID is not for this study though it includes some of the authors. I did attempt to find the correct entry but could not find it.

Introduction

1. This is well structured and concise, however it could do with more detail as ooutlined below.

2. There are a number of statements made that are not backed up by appropriate references (last sentence of paragraph 1, 3rd sentence of paragraph 2, and 1st sentence of paragraph 3)

3. The authors' argument is generally good however they have not sufficiently presented on previous reviews into screening tools and outcome measures. There has certainly been reviews on this before as can be seen from a quick google scholar search, though perhaps screening in educational settings is sufficiently niche that it is an area that does need exploring. However, by not reviewing previous reviews it makes it seem that the authors do not adequately know the literature nor have they sufficiently argued for the importance of this review to cover what previous reviews have not.

Methods

1. Structured well and everything is clear and in the correct sections but lacks detail.

2. AS stated previously the PROSPERO ID is incorrect.

3. The authors used a sufficient number of databases but I feel the search strategy missed some important key terms. This is especially true for educational settings, which only include variations on school, pupil, student, and classroom; particular because the authors are taking a worldwide perspective. For example variations on education, tutelage and college should at least be added. Similarly variations of neurodiversity and Aspergers Syndrome should be included. Without these I don't believe the searches are expansive enough and the results are unlikely to be comprehensive.

4. More detail is needed for the selection criteria generally. The article later mentions special schools were excluded for example but this is not listed in this section. Details of which types of studies are excluded/included are similarly vague. For example, the authors say primary research studies are excluded but that is not accurate.

5. No justifications are given for why certain types of evidence are excluded such as conference abstracts and grey literature.

6. No supplementary searches were conducted such as the reference lists of included studies or contacting authors. One thing in particular which should have been done is examing the

manuals of the screening tools which will normally list the studies that have been conducted on their tool validation.

7. References for software are missing such as Covidence and Excel.

8. The synthesis section states that a narrative synthesis was to be conducted. This is somewhat inconsistent with the results which state that not enough data was provided for a meta-analysis so a narrative synthesis was conducted. The method section should indicate that this was planned if possible.

Results

1. The results indicate that manual searching did not yield further studies. The report does not say what was manually searched here or in the methods however.

2. The PRISMA diagram is inaccurate as the totals do not add up correctly (e.g. 3788+185=3973). Also it indicates 142 studies were included; the non-ASD studies should be listed in the excluded studies section.

3. There is not enough detail on the study selection results in the text. Hence, the errors in the PRISMA can't be explained. Similarly, there appears to have been an update conducted but no detail is provided on shat if any studies were added as a result of the updaete.

4. The actual summary of the findings was fine but I don't have confidence in them due to previous issues.

Discussion

1. Again this was fine but there are some typos and statements without appropriate references.

Apologies if this review appears overly harsh as I reiterate that I think this study was well conducted. It just needs to be a bit more expansive and for more time to be spent on editing this manuscript.

3.

Introduction missing references and doesn't adequately cover the extent of the literature.

Prospero ID not correct.

Search is not adequate.

No justifications for criteria

Descrtiption of process not complete

PRISMA inaccurate

Description of results not complete

Typos in discussion

Criteria not clear

VERSION 1 - AUTHOR RESPONSE

**Reviewer: 1**<br>**Dr. Maria Bujnowska-Fedak, Wroclaw Medical University**	-
1. The number of identified school-based ASD screening tools is different in the abstract, where seven tools are mentioned, and in the main text, where eight tools were identified and analyzed. This requires correction.	Thank you, this has been corrected in the abstract [page 2, "results" section]
2. The discussion needs to be expanded and take on a broader perspective. It also seems inconsistent and contradicts earlier obvious statements in some parts.<br>In Discussion (age 15) in lines 51-56 there is a sentence: 'Importantly, it remains unclear whether early detection through screening improves long-term outcomes for individuals living with ASD'. Do you really agree with this statement? There is strong evidence that early diagnosis of ASD is crucial and can positively impact a child's later development.<br><br>For example, look at the references below:<br>Smith T, Klorman R, Mruzek DW. Predicting Outcome of CommunityBased Early Intensive Behavioral Intervention for Children with Autism. J Abnorm Child Psychol. 2015;43:1271–82.<br>Orinstein AJ, Helt M, Troyb E, Tyson KE, Barton ML, Eigsti IM, et al. Inter- vention for optimal outcome in children and adolescents with a history of Autism. J Dev Behav Pediatr. 2014;35:247–56.<br>MacDonald R, Parry-Cruwys D, Dupere S, Ahearn W. Assessing progress and outcome of early intensive behavioral intervention for toddlers with autism. Res Dev Disabil. 2014;35:3632–44.<br>Sobieski, M., Sobieska, A., Sekułowicz, M. et al. Tools for early screening of autism spectrum disorders in primary health care – a scoping review. BMC Prim. Care 23, 46 (2022). https://doi.org/10.1186/s12875-022-01645-7.<br><br>Furthermore, this statement contradicts your previous statement in the Introduction (page 5, lines 42-44): 'Screening enables earlier evidence-based interventions that can help prevent the negative effects of a delayed ASD diagnosis, ultimately improving individuals' quality of life'.	Thank you for highlighting this. We agree that early diagnosis is strongly associated with improved developmental outcomes and quality of life. Our intention in the discussion was not to contradict this, but reflect on the uncertainty within the literature on whether a universal screening programme (as opposed to case-finding/ targeted referral) was able to deliver these benefits. We have rephrased these in the introduction [page 4, paragraph 3] and discussion [page 16, paragraph 3] to ensure this is clear. We have also included these references, thank you.
**Reviewer: 2**<br>**Mr. David Marshall, Lime Trees CAMHS**	-

**Abstract**	
1. I actually think this is well written for the most part. My only concern is that the PROSPERO registration ID is not for this study though it includes some of the authors. I did attempt to find the correct entry but could not find it.	Thank you. Apologies, this PROSPERO registration was included by mistake. This review was initiated as a scoping review rather than a systematic review with a prespecified protocol, and therefore was not eligible for PROSPERO registration. We have now clarified this in the methods section to avoid confusion. [page 6, line 5]
**Introduction**	
This is well structured and concise; however it could do with more detail as outlined below.<br>1. There are a number of statements made that are not backed up by appropriate references (last sentence of paragraph 1, 3rd sentence of paragraph 2, and 1st sentence of paragraph 3)	Thank you, we have included more discussion of the existing literature around screening, including the gap in knowledge that our review addresses, and included additional references on screening tools and outcome measures to introduction and discussion [page 4, paragraph 3] and [page 14, paragraph 3].
2. The authors' argument is generally good however they have not sufficiently presented on previous reviews into screening tools and outcome measures. There has certainly been reviews on this before as can be seen from a quick google scholar search, though perhaps screening in educational settings is sufficiently niche that it is an area that does need exploring. However, by not reviewing previous reviews it makes it seem that the authors do not adequately know the literature, nor have they sufficiently argued for the importance of this review to cover what previous reviews have not.	Thank you, we have added some additional references on screening tools and outcome measures to introduction and discussion [page 4, paragraph 3] and [page 16, paragraph 3].
**Methods**	
1. Structured well and everything is clear and in the correct sections but lacks detail. AS stated previously the PROSPERO ID is incorrect.	Thank you. Apologies, this PROSPERO registration was included by mistake. This review was initiated as a scoping review rather than a systematic review with a prespecified protocol, and therefore was not eligible for PROSPERO registration. We have now clarified this in the methods section to avoid confusion [page 7, line 5].
2. The authors used a sufficient number of databases but I feel the search strategy missed some important key terms. This is especially true for educational settings, which only include variations on school, pupil, student, and classroom; particular because the authors are taking a worldwide perspective. For example variations on education, tutelage and college should at least be added. Similarly variations of neurodiversity and Aspergers Syndrome should be included. Without these I don't believe the searches are expansive enough and the results are unlikely to be comprehensive.	Thank you for this helpful suggestion. We acknowledge that our search strategy could have included additional variations of educational terms (e.g., education, tutelage, college) and neurodiversity-related terms (e.g., Asperger's Syndrome, neurodiversity). We have added this as a limitation in the manuscript.<br><br>We also note that the majority of evidence on population-level screening for medical conditions is published in medical and public health journals rather than educational databases. Including additional educational databases would substantially increase screening time and resource requirements, with relatively limited impact on identifying studies relevant to population screening for ASD. [page 18, paragraph 1]

3. More detail is needed for the selection criteria generally. The article later mentions special schools were excluded for example but this is not listed in this section. Details of which types of studies are excluded/included are similarly vague. For example, the authors say primary research studies are excluded but that is not accurate.	Thank you, we have removed "primary research" from the exclusion criteria as this was a mistake. [page 8, paragraph 2] and added to the selection criteria.
4. No justifications are given for why certain types of evidence are excluded such as conference abstracts and grey literature.	Thank you. We excluded these to ensure that all included evidence was peer-reviewed, accessible in full text, and had sufficient information on methods and outcomes. We have added this into the methods section for clarity. [page 7, paragraph 2, line 4]
5. No supplementary searches were conducted such as the reference lists of included studies or contacting authors. One thing in particular which should have been done is examing the manuals of the screening tools which will normally list the studies that have been conducted on their tool validation.	Thank you – we acknowledge that we did not conduct supplementary searches such as reference list screening, contacting authors, or reviewing the manuals of individual screening tools. Our approach was to focus on systematic database searching to ensure transparency and reproducibility within the resources available. We agree, however, that additional approaches such as consulting tool manuals could have identified further relevant studies, and we have now noted this as a limitation in the Discussion section. [page 18, line 1]
6. References for software are missing such as Covidence and Excel.	Thank you, we have added in these references. [page 8, paragraph 1, line 1] and [page 8, paragraph 2, line 1]
7. The synthesis section states that a narrative synthesis was to be conducted. This is somewhat inconsistent with the results which state that not enough data was provided for a meta-analysis so a narrative synthesis was conducted. The method section should indicate that this was planned if possible.	Thank you for this helpful point. To clarify, this review was designed as a scoping review from the outset, with narrative synthesis as the planned method of data synthesis. We did not intend to conduct a meta-analysis, as our objective was to map and describe the existing evidence rather than quantify pooled effects. We have revised the Methods section to better reflect this intention. [page 7, paragraph 1]
**Results**	
1. The results indicate that manual searching did not yield further studies. The report does not say what was manually searched here or in the methods however.	Thank you and we agree this is not clear. We have added to the methods that we did not do additional manual searches and have also included this in the limitations for clarity. [page 18, line 2] and [page 7, paragraph 3, line 6]
2. The PRISMA diagram is inaccurate as the totals do not add up correctly (e.g. 3788+185=3973). Also it indicates 142 studies were included; the non-ASD studies should be listed in the excluded studies section.	Thank you, we have adjusted the PRISMA diagram to ensure the line numbers is accurate. [figure 1]
3. There is not enough detail on the study selection results in the text. Hence, the errors in the PRISMA can't be explained. Similarly, there appears to have been an update conducted but no detail is provided on shat if any studies were added as a result of the update.	Thank you, we have adjusted the PRISMA diagram to ensure the line numbers is accurate. [figure 1]
**Discussion**	
1. Again, this was fine but there are some typos and statements without appropriate references.	Thank you, we have addressed these throughout and left in highlight.

2.	Apologies if this review appears overly harsh as I reiterate that I think this study was well conducted. It just needs to be a bit more expansive and for more time to be spent on editing this manuscript.	Thank you for your comments, we agree and have now addressed them.
3.	Introduction missing references and doesn't adequately cover the extent of the literature.<br>Prospero ID not correct.<br>Search is not adequate.<br>No justifications for criteria<br>Descrtiption of process not complete<br>PRISMA inaccurate<br>Description of results not complete<br>Typos in discussion<br>Criteria not clear	These should be addressed in above comments.
